# Using structural equation modelling to untangle sanitation, water and hygiene pathways for intervention improvements in height-for-age in children <5 years old

**DOI:** 10.1093/ije/dyz202

**Published:** 2019-10-09

**Authors:** Heather Reese, Sheela S Sinharoy, Thomas Clasen

**Affiliations:** 1 Rollins School of Public Health, Emory University, Atlanta, GA, USA; 2 London School of Hygiene and Tropical Medicine, London, UK

**Keywords:** Water and sanitation, hygiene, stunting, India, path analysis

## Abstract

**Background:**

Despite a strong theoretical rationale for combining water, sanitation and hygiene (WaSH) interventions to improve child health, study findings are heterogeneous with little understanding of the mechanisms for these effects. Our study objective was to demonstrate the utility of structural equation modeling (SEM) to assess intervention effects on height-for-age *z* score (HAZ) through the complex system of WaSH pathways.

**Methods:**

We used data from a matched cohort effectiveness evaluation of a combined on-premise piped water and improved sanitation intervention in rural Odisha, India. Height/length was measured in children 0–59 months old (*n* = 1826) from 90 matched villages in February–June 2016. WaSH behaviours and infrastructure were assessed through household surveys and observation, respectively. We used SEM to calculate the standardized path coefficients and the total contributions of WaSH pathways to HAZ.

**Results:**

Intervention improvements on HAZ were through the sanitation pathway (coverage → use β: 0.722; use → HAZ β: 0.116), with piped water coverage indirectly affecting HAZ through improved sanitation use (β: 0.148). Although the intervention had a positive association with handwashing station coverage, there was no evidence of a total hygiene pathway effect on HAZ or further direct effects through the water pathways.

**Conclusions:**

This study demonstrates the utility of SEM to assess the mechanisms through which combined WaSH interventions impact HAZ as a system of pathways, providing a more nuanced assessment than estimation of the total intervention effect. Our finding, that water impacts HAZ through the sanitation pathway, is an important and actionable insight for WaSH programming.

**Key Messages**

- There is a strong theoretical rationale for combining water, sanitation and hygiene (WaSH) interventions to improve child health, although findings on the impact of combined interventions are heterogenous.
- Risk factors for linear growth faltering have been well characterized, but little is known about the complex system of pathways that mediates WaSH effects on child height-for-age *z* score (HAZ).
- In our study, sanitation was the primary pathway for intervention improvements in child HAZ. The positive association between on-premise piped water coverage and HAZ was mediated by improvements in sanitation use, but remaining water pathways had no effect on HAZ, nor did the hygiene pathway.
- Future programmes may consider including on-premise water coverage with sanitation in combined interventions to improve sanitation use, even in the absence of potential direct effects of water on health.

## Introduction

In 2015, the Sustainable Development Goals (SDGs) reaffirmed the global community’s commitment to ensuring core development and health standards for all people, including ensuring safe water and sanitation, as well as improvements in child health.^1^ Although the association between water, sanitation and hygiene (WaSH) conditions and child health are well established, the effectiveness of WaSH interventions for improving child health and nutrition, as measured by height-for-age *z*-scores (HAZ), has been highly variable by study and setting.^2–8^ In addition, recent meta-analyses provided evidence that WaSH interventions, singly or combined, improved HAZ by ∼0.08.^9^^,^^10^ Most of this evidence is from observational studies, with substantial differences in intervention implementation, type of single or combined WaSH intervention, as well as in study setting.

Recent randomized controlled trials provide a complement to the current body of primarily observational evidence. An evaluation of a community-led total sanitation intervention in Mali reported substantial improvements in child HAZ associated with the intervention.^5^ In contrast, the WASH Benefits trial in Bangladesh and Kenya, which assessed the impact of water, sanitation, handwashing and child nutrition improvements, found no effect of any combination of WaSH components on child HAZ.^6^^,^^7^ Similarly, the Sanitation, Hygiene, Infant Nutrition Efficacy (SHINE) study in Zimbabwe also observed no effect of the WaSH intervention on child linear growth.^8^ Taken together, the evidence demonstrates the continuing uncertainty, not only about the potential effects of WaSH interventions but also the mechanisms through which WaSH interventions may impact child linear growth.^11^

The physiological mechanisms linking WaSH and child linear growth are hypothesized to operate primarily through diarrhoea and/or environmental enteric dysfunction (EED). Poor WaSH conditions increase the risk of diarrhoea, which can lead to impaired linear growth through multiple pathways including reduced energy intake, nutrient loss and malabsorption.^12^^,^^13^ EED is a subclinical disorder common among individuals living with poor WaSH conditions. These conditions and the resulting persistent exposure to enteropathogens are hypothesized to lead to blunting and atrophy of the villi of the small intestine, causing nutrient loss, malabsorption and intestinal and systemic inflammation, and in turn leading to linear growth impairment.^8^^,^^14^

Several hypotheses exist to explain the lack of observed effects on child linear growth in recent trials of WaSH interventions. A primary hypothesis is that measurable improvements in child health require complete or almost complete interruption of multiple intersecting pathways. Findings of no discernable improvements may be due to insufficient interruption of pathways or an incomplete understanding of the primary pathways. In addition, the relationship between health and WaSH may be non-linear: as a population progresses up the respective water, sanitation and hygiene ladders, there may be incrementally smaller gains in health. Another hypothesis is that the common circulating pathogens responsible for child disease burden are setting specific, both geographically and temporally, and thus may require interventions tailored to the dominant pathways within each setting.

The evidence for WaSH and health focuses on estimating the main effects of an intervention on health outcomes that are causally several steps removed from the intervention being evaluated. Studies often measure some intermediate outcomes, such as measures of the quality of programme implementation, including coverage and availability of infrastructure, with fewer studies reporting prevalence of WaSH behaviours, microbiological quality of drinking water or faecal contamination of the household environment. However, these intermediate outcomes are often only assessed descriptively. Understanding intervention effects as a system of intersecting pathways may provide needed additional evidence for policy making and programme development.

Structural equation modelling (SEM) has been employed throughout the behavioural health sciences, but to our knowledge it has not been used to assess the system of pathways for combined WaSH interventions. Path analysis helps assess the theorized intersecting pathways of intervention effect that motivate programme design, and relies on a strong theoretical framework. Assessing the paths of intervention effects through intermediate outcomes, not just the total effect on health, may provide needed additional evidence by allowing the assessment of effects for the individual water, sanitation and hygiene pathways in addition to the system of pathways, and aid in programme development and modification by identifying ‘leaky’ pathways.

We demonstrate the utility of this approach through the path analysis of the effects of a matched cohort evaluation of a combined community-level sanitation and on-premise piped water intervention implemented by Gram Vikas, in Odisha, India on child HAZ. Previous assessment of the main effects of this combined intervention found it was associated with improvements in child HAZ [+0.17, 95% confidence interval (CI): 0.03–0.31].^15^ Our objectives were to: (i) assess whether WaSH infrastructure coverage, availability and use behaviours mediate the relationship between this combined intervention and child HAZ, and (ii) compare the direct and indirect associations of this WaSH intervention with child HAZ using SEM. The analysis focuses on child HAZ because of its global importance as a marker of child nutritional status. Unlike other markers, such as weight-for-age *z*-score or weight-for-height *z*-score, HAZ is a marker of a child’s exposure to nutritional and environmental factors over the long-term, making it appropriate for our matched cohort study.^16^

## Methods

### Study design, intervention and participants

This study is part of a matched cohort evaluation to assess the effectiveness of a water and sanitation intervention in rural Ganjam and Gajapati districts within Odisha, India. The MANTRA programme (Movement and Action Network for the Transformation of Rural Areas) was implemented by the Indian NGO, Gram Vikas. The intervention consisted of: (i) a household pour-flush toilet with dual soak-away pits, (ii) an attached bathing room, and (iii) household piped water connections in the toilet, bathing room and the kitchen. Access to the piped water system was contingent on full community coverage of household toilets. Further intervention details have been previously described.^17^

Forty-five intervention villages were randomly selected from a list, provided by Gram Vikas, of villages where the intervention was implemented in Ganjam and Gajapati districts. Forty-five control villages were matched to the selected intervention villages through a restriction, matching and exclusion process; matching was effective in balancing the intervention and control study arms.^17^ Whereas the matched cohort study collected data over four rounds, data used in this analysis were collected in a single round in February–June 2016. Households with a child <5 years of age were eligible for enrollment, up to 40 households per village were enrolled and anthropometry was measured in available children <5 years old (*n* = 1826). Complete information on all variables included in the analysis were available for 1206 children.

The male and/or female head of the household provided written informed consent for the household. The study was reviewed and approved by the Ethics Committee of the London School of Hygiene and Tropical Medicine, U.K (No. 9071) and Institute Ethics Committee of the Kalinga Institute of Medical Sciences of KIIT University, Bhubaneswar, India (KIMS/KIIT/IEC/053/2015).

### Measurements

#### Anthropometry

Recumbent length and height were measured using standard anthropometric methods.^18^^,^^19^ Recumbent length was measured to the nearest 0.1 cm for children <2 years old using a portable length board (Seca 417; Seca, Birmingham, UK). Standing height was measured to the nearest 0.1 cm for children 2–5 years old using a stadiometer (Seca 213). Height/length were collected in duplicate, and if measurements differed by >0.7 cm, a third was collected; the mean of measurements was used to calculate *z*-scores according to WHO 2006 growth standards (R igrowup macro).^20^ Back-checks on height/length were conducted on a randomly selected 10% of households.

#### Water, sanitation and hygiene mediating variables

Household surveys were administered to the primary caregiver in Odia and collected data on household sociodemographic characteristics, infrastructure and reported household and individual behaviours. In addition, field workers conducted spot-check observations of water, sanitation and hygiene infrastructure and conditions. Improved sanitation coverage (flush/pour flush to piped sewer system, septic tank or pit latrine; ventilated improved pit latrine; composting toilet; pit latrine with slab) was defined according to the Joint Monitoring Programme (JMP) standard definition.^21^ Usual defecation location was self-reported for the following categories within each household: elders ≥60 years, men 18–59 years, women 18–59 years and children 5–17 years. For children <5 years old, the caregiver reported the disposal location for the child’s last defecation event, and improved child faeces disposal was defined as disposal into an improved toilet. From these binary defecation location or faeces disposal variables, we calculated household sanitation use as the proportion of household members reporting improved toilet use for defecation (members >5 years old) or for child faeces disposal (members ≤5 years old) out of the total number of members within each household. Piped water coverage was defined as a piped water source located on the household premises. Drinking water storage was defined as no storage, safe storage in a covered narrow mouthed (<6 cm diameter) container or unsafe storage. Presence of a handwashing station was defined as a designated location with both water and cleansing agent available, according to the JMP standard definition. Reported availability for the preferred drinking water source was assessed using two measures: (i) source unavailable for ≥24 h in the previous 2 weeks, and (ii) source unavailable at any time in the previous 24 h. Water source availability was categorized as any interruption, using either measure.

#### Confounders

Potential confounders were determined through correlation with the intervention and anthropometric measurements, and through review of the literature. Covariates included female caregiver education (0–5 completed years of schooling, >5 completed years of schooling), household caste/tribe (scheduled caste, scheduled tribe, other backward caste, other caste), household food insecurity (little to no hunger, or moderate to severe hunger in the household, as measured using the household hunger scale^22^), livestock ownership (ownership of any poultry, small or large livestock), child minimum dietary diversity and standardized household wealth index. Minimum dietary diversity was calculated as at least four food groups consumed by the child over the past 24 h. The household wealth index was derived using principal components analysis (R psych package, version 1.6.12) including household asset ownership (chair, table, refrigerator, mattress, pressure cooker, scooter or motorcycle, mobile phone, electric fan, sewing machine and television), housing characteristics, agricultural land acreage owned and below poverty-line status.^23–25^

### Statistical analysis

**Figure 1.**
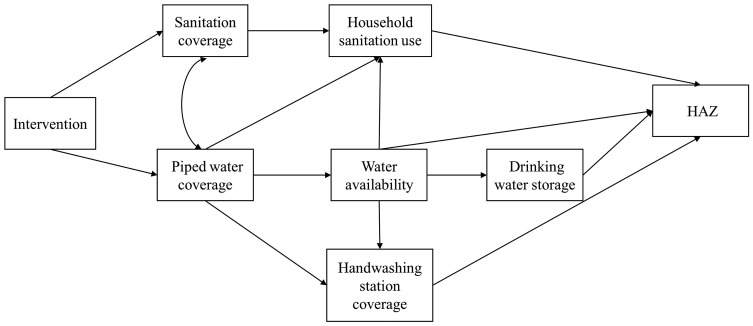
Conceptual model describing the hypothesized relationships between intervention status, improved sanitation coverage, household sanitation use, on-premise piped water coverage, reported interruption in water availability, household drinking water storage, handwashing station coverage and height-for-age *z*-score (HAZ).

The intervention theory of change was used to describe the complex system of water, sanitation and hygiene pathways and the theorized impact on child growth (Figure 1). Since the intervention was implemented as a combined intervention, sanitation coverage and on-premise piped water coverage were allowed to covary.

Structural equation modelling (R lavaan and lavaan.survey packages, versions 0.5–23.1097 and 1.1.3.1) was used to simultaneously fit this system of multiple paths as one model, adjusting for the hierarchical structure of the data with children nested within villages.^26^^,^^27^ Path analysis using SEM relies on a strong theoretical framework to inform the hypothesized system of pathways. Therefore, it is primarily a confirmatory technique to assess whether the proposed model is supported by the data and relies on correct specification of the direction of causal relationships within the model.

We calculated the standardized coefficients for each path, as well as the standardized total associations of the intervention with HAZ. In addition, we calculated the standardized indirect effects for each of the sanitation, water and hygiene pathways. We assessed model robustness by testing the categorization of mediating variables for water storage and handwashing station coverage, and the inclusion of a remaining direct path from the intervention. All analyses were conducted in R (version 3.4.2).^28^

## Results

Table 1 presents descriptive data on the study population. A higher proportion of children in intervention villages had caregivers with at least primary schooling (55% vs 47%) and were in the richest households (23% vs 13%) than children in the control. A smaller proportion of intervention children (12%) than control children (23%) were members of scheduled castes. The majority of intervention children had access to improved sanitation facilities (82%), on-premise piped water sources (64%) and a handwashing station with soap and water available (86%). The majority of both intervention and control children lived in households that stored drinking water, with only about 20% using a safe storage method. Child’s HAZ was positively associated with village intervention status, though on average children in both intervention and control villages were more than one standard deviation below the population average HAZ.

**Table 1.** Characteristics of the study population, by intervention and control village status

	Control *n* = 994	Intervention *n* = 832	*P*-value^a^
Household characteristics			
Caregiver education ≥primary school, *n* (%)	465 (46.8%)	460 (55.3%)	0.066
Caste/tribe, *n* (%)			0.009
Scheduled caste	199 (23.8%)	99 (13.8%)	
Scheduled tribe	149 (17.8%)	115 (16.0%)	
Other backward caste	302 (36.2%)	271 (37.6%)	
Other caste	185 (22.2%)	235 (32.6%)	
Wealth index quintile, *n* (%)			0.018
Poorest	243 (27.4%)	127 (16.9%)	
Poor	167 (18.8%)	152 (20.2%)	
Middle	191 (21.6%)	142 (18.9%)	
Rich	171 (19.3%)	157 (20.9%)	
Richest	114 (12.9%)	173 (23.0%)	
On-premise piped water, *n* (%)	86 (8.7%)	534 (64.2%)	<0.001
Improved toilet, *n* (%)	183 (18.4%)	682 (82.3%)	<0.001
No interruption in water availability, combined *n* (%)	899 (90.4%)	656 (78.8%)	<0.001
No interruption in previous 2 weeks	946 (95.2%)	720 (86.5%)	<0.001
No interruption in previous 24 h	910 (91.5%)	705 (84.7%)	<0.001
Handwashing station, *n* (%)	467 (61.1%)	565 (84.6%)	<0.001
Proportion of household members using improved sanitation	0.11 (0.27)	0.55 (0.37)	<0.001
Drinking water storage, *n* (%)			<0.001
No storage	4 (0.4%)	24 (2.9%)	
Safe storage (narrow mouth, covered container)	222 (22.4%)	168 (20.3%)	
Unsafe storage	766 (77.2%)	635 (76.8%)	
Household food insecurity, *n* (%)			0.484
No to little hunger	754 (96.4%)	650 (97.5%)	
Moderate to severe hunger	26 (3.3%)	17 (2.5%)	
Minimum dietary diversity, *n* (%)	509 (54.6%)	479 (60.6%)	0.116
Livestock ownership, *n* (%)	353 (41.5%)	305 (42.9%)	0.882
Child characteristics			
Age, months	31.9 (16.3)	32.1 (16.0)	0.787
Sex, female *n* (%)	515 (51.8%)	415 (49.9%)	0.438
HAZ	−1.77 (1.12)	−1.48 (1.17)	0.009

a *P*-values adjusted for clustering at the village level.

**Figure 2.**
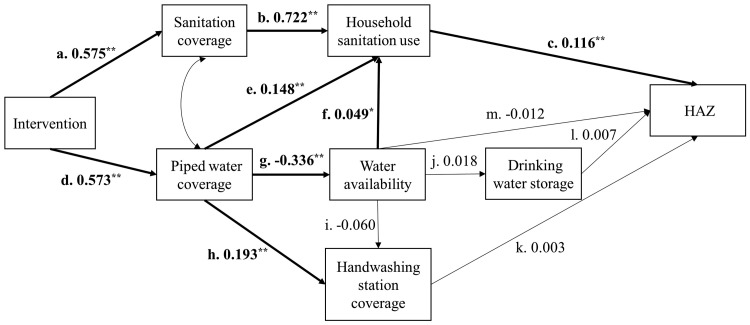
Structural equation model with standardized path coefficients for the relationships among intervention status, improved sanitation coverage, household sanitation use, on-premise piped water coverage, reported interruption in water availability, household drinking water storage, handwashing station coverage, and height-for-age *z*-score (HAZ). Bold lines indicate significant associations (* *P* < 0.05, ** *P* < 0.01).

Figure 2 presents the path diagram with standardized coefficients for each path; Table 2 presents indirect and total effects for WaSH pathways calculated from unstandardized and standardized coefficients. Use of standardized coefficients provides a simpler comparison of path coefficients from variables measured on different scales within the same model, and so standardized coefficients are presented in the text hereafter.

**Table 2.** Indirect and total associations of the intervention, sanitation coverage, on-premise piped water coverage and handwashing station coverage with child height-for-age *z* score^a^

	Unstandardized coefficient (95% CI)	Standardized coefficient (95% CI)	*P*-value
Sanitation pathway (b → c)			
Total association	0.196 (0.088, 0.304)	0.084 (0.038, 0.129)	<0.001
Water pathways			
Water 1 (e → c)	0.041 (0.010, 0.073)	0.017 (0.004, 0.000)	0.011
Water 2 (g → f → c)	−0.005 (−0.010, 0.073)	−0.002 (−0.004, 0.000)	0.103
Water 3 (g → m)	0.010 (−0.054, 0.074)	0.004 (−0.023, 0.031)	0.762
Water 4 (g → j → l)	0.000 (−0.002, 0.002)	0.000 (−0.001, 0.001)	0.899
Water 5 (g → i → k)	0.000 (−0.003, 0.004)	0.000 (−0.001, 0.002)	0.936
Water 6 (h → k)	0.001 (−0.032, 0.035)	0.001 (−0.013, 0.014)	0.935
Total association	0.048 (−0.020, 0.117)	0.020 (−0.008, 0.048)	0.168
Hygiene pathway (k)			
Total association	0.008 (−0.181, 0.197)	0.003 (−0.069, 0.075)	0.936
Intervention (sanitation and water pathways)			
Total association	0.139 (0.060, 0.218)	0.060 (0.026, 0.093)	0.001

a Adjusted for household wealth, caste/tribe, caregiver’s education, household food insecurity, livestock ownership and minimum dietary diversity.

In the SEM model, there was a similar magnitude positive association between the intervention and improved sanitation coverage [β: 0.575, 95% CI: 0.194–0.365; path a] and on-premise piped water coverage (β: 0.573, 95% CI: 0.452–0.694; path d) (Figure 2). Coverage of improved sanitation was also positively associated with the proportion of the households using an improved toilet for defecation (β: 0.722, 95% CI: 0.658–0.785; path b), as was household sanitation use on child HAZ (β: 0.116, 95% CI: 0.052–0.180; path c). However, on-premise piped water coverage was negatively associated with water availability (β: −0.336, 95% CI: −0.466, −0.207; path g), and although water availability showed a small positive association with household sanitation use, no further water associated paths downstream of water availability were associated with child HAZ.

Overall, the sanitation pathway, from improved sanitation coverage to household use (path b → c), had the strongest positive effect on HAZ (β: 0.084, 95% CI: 0.038–0.129) (Table 2). The water pathway was conceptualized as the combination of several pathways. On-premise piped water coverage had an indirect positive effect on HAZ through increases in household sanitation use (β: 0.017, 95% CI: 0.004–0.030; path e → c), but there was no evidence of an indirect effect on sanitation use through increased water availability (path g → f → c). Piped water coverage decreased availability of drinking water (β: −0.336, 95% CI: −0.466, −0.207; path g), and water availability was neither directly associated with HAZ (path m) nor indirectly associated through drinking water storage (path j → l). Although on-premise piped water coverage had a positive effect on availability of a handwashing station within the household (β: 0.193, 95% CI: 0.119–0.268; path h), the hygiene pathway (path k), described as a path within the water pathway given the reliance on water availability, had no effect on HAZ.

The following sensitivity analyses were used to assess the robustness of the model: (i) re-categorizing water storage (no storage vs any storage), and (ii) re-categorizing handwashing station coverage (water and soap/detergent available vs any other). For water storage and handwashing station coverage, there was a qualitatively negligible difference in estimates, regardless of variable categorization.

We also assessed for a remaining direct pathway from the intervention to HAZ. However, with an estimate at zero, there was no evidence of an omitted mediator in this direct pathway. This substantiates the underlying theoretical framework that the intervention effect on HAZ is mediated through the WaSH pathways, conditioned on household wealth, caste/tribe, caregiver’s education, household food insecurity, livestock ownership and minimum dietary diversity.

Model fit statistics for all structured equation models met the respective standard cut-off values (*P* > 0.05, <0.05, >0.95, >0.95 and <0.08 for the χ^2^ test, root mean square error of approximation, comparative fit index, Tucker-Lewis index and standardized root mean square residual, respectively).^29^^,^^30^

## Discussion

This study demonstrates the utility of a systems approach for assessing the complex associations between WaSH improvements and health through analysis of the water, sanitation and hygiene pathways. Within our study population, intervention effects on child HAZ were mediated through the sanitation pathway (path a → b → c), with intervention improvements in on-premise piped water access indirectly affecting HAZ through association with improved sanitation use (path d → e → c). There was no evidence that effects on water availability, an indicator of water quantity, or water storage, an indicator of drinking water quality, were associated with HAZ. Our findings also showed no evidence that the hygiene pathway was associated with HAZ.

Although we found no evidence of a possible omitted mediator in the direct intervention pathway, a further theoretical possibility is a remaining mediating pathway through dietary intake.^9^^,^^31^ This pathway would be expected if the on-premise piped water access allowed households to grow a greater diversity of micronutrient-rich fruit and vegetable crops or to raise a larger number of livestock for consumption of animals or animal products.^32^ In Sub-Saharan Africa, both access to irrigation water for household food production and decreased water collection time have been shown to be associated with nutrition.^33^ However, previous research within our study population showed no evidence that the intervention was associated with dietary diversity in children aged 6–23 months, or with household crop production, poultry ownership or livestock ownership.^34^ This further strengthens the findings that the intervention effects on HAZ were through WaSH infrastructure improvements, dominated by the sanitation pathway.

These findings suggest that WaSH programmes that provide sanitation infrastructure may also benefit from the provision of on-premise piped water. Other studies in India have observed a strong preference for pour-flush toilets, which require water for flushing; without this programme component, households may be less likely to use sanitation infrastructure. We hypothesize that, in our study setting, the presence of on-premise piped water led to an increased use of toilets in intervention households, resulting in reduced child exposure to enteric pathogens and an improvement in child linear growth. However, it is important to note that these findings are not broadly generalizable. The contributions of on-premise piped water through improving toilet use may only be relevant to similar settings in which pour-flush toilets, or other toilets reliant on water, are the preferred sanitation infrastructure.

This study has several strengths. To our knowledge, this is the first use of the SEM approach to estimate the effects of a combined WaSH intervention on HAZ as a complex intersecting system of pathways. In this case, SEM provides substantial benefits over classic regression through simultaneously modelling the system of paths, avoiding multiple individual tests of significance if each pathway were instead modelled separately. Although measurements of some mediating variables relied on self-reports, e.g. defecation behaviour and interruptions in water availability, and are thus subject to measurement bias, all other variables were directly observed or otherwise measured.^35^ However, it is important to note that pathways cannot be interpreted as causal; this analysis used data collected at the same time point HAZ was assessed.

Additionally, this study assessed a simplified model system of WaSH pathways. A more nuanced assessment of specific pathways, such as explicit measurement of hygiene behaviours, could provide more targeted programmatic recommendations.^36^ In addition, we chose to focus on mediating variables at the household level, although strong evidence exists for the interdependence of individual and community water and sanitation characteristics.^37–39^ Further analysis is needed to assess the relative importance and interdependence of individual, household and community level mediators on child health.^40^ Finally, this analysis does not include measures of microbiological source and drinking water quality as mediators. Future analyses would benefit from the inclusion of these more objective measures, as well as a more integrated systems approach to assessment of which microbiological measures mediate the association between WaSH infrastructure improvements and child health.

In conclusion, using an SEM approach to estimate the effectiveness of WaSH interventions as a system of integrated pathways allows a more nuanced assessment and may provide more direct programmatic relevance. Although the parent study found that the intervention was associated with improvements in HAZ using a classic regression approach, it was not able to assess how these effects may have occurred. Our findings from this analysis supplement our previous findings and suggest that the intervention effect on improving HAZ primarily acted through increasing improved sanitation use for defecation, and that any intervention effects on improving water or hygiene were vitiated prior to impacting HAZ. This also underlies the importance of a combined WaSH approach, even when water may not directly benefit health. Future assessment of the effectiveness of combined WaSH interventions may benefit from incorporating a path analytic framework in addition to estimation of total effects, to match analysis to the underlying motivating theory and provide more targeted programmatic recommendations.

## Author Contributions

T.C., H.R. and S.S. contributed to study design. H.R. completed the analyses. All authors contributed to editing and revising the manuscript.

## Funding

This work was supported by the Bill & Melinda Gates Foundation to the London School of Hygiene & Tropical Medicine [grant number OPP1008048] and by Emory University [grant number OOP1125067].

**Conflict of interest:** None declared.
